# Validation of the Polish version of the Johns Hopkins Learning Environment Scale–a confirmatory factor analysis

**DOI:** 10.1038/s41598-024-61391-x

**Published:** 2024-05-12

**Authors:** Dorota Wójcik, Leszek Szalewski, Adam Bęben, Iwona Ordyniec-Kwaśnica, Robert B. Shochet

**Affiliations:** 1 Department of Dental Prosthetics, Medical University of Lublin, Lublin, Poland; 2 Digital Dentistry Lab, Department of Dental and Maxillofacial Radiodiagnostics, Medical University of Lublin, Lublin, Poland; 3 Department of Dental Prosthetics, Medical University of Gdańsk, Gdańsk, Poland; 4 Department of Medicine, Albert Einstein College of Medicine, New York City, NY USA

**Keywords:** Medical education, JHLES questionnaire, Dental students, Learning environment, Medical research, Health care

## Abstract

The Johns Hopkins Learning Environment Scale (JHLES) was developed by Robert B. Shochet, Jorie M. Colbert and Scott M. Wright of the Johns hopkins university school of medicine and consists of 28 items used to evaluate perception of the academic environment. The objective was to translate and adapt the JHLES to Polish cultural conditions and to validate the Polish version of the tool. The JHLES questionnaire was completed by students of all years (first–fifth) of the faculties of dental medicine at the Medical University of Lublin and the Medical University of Gdańsk. The total surveyed population consisted of 597 students. The overall reliability of the tool was excellent. Confirmatory factor analysis was performed in order to confirm structural consistency with the original JHLES tool. Consequently, all indices had acceptable values (close to 1 or 0, depending on the case), and there was consistency in the results, which shows that the JHLES model is supported by the data. In the present study, the JHLES has been validated in a sample of dental students for the first time in Poland and Europe. Our study provided good evidence for the reliability and validity of the Polish version of the JHLES. In conclusion, the Polish-language version of the JHLES questionnaire is a reliable and valid instrument for analysing the learning environment for students, and its factor structure is supported by the data.

## Introduction

Recently many authors have witnessed a trend toward increased attention being paid to the learning environment-related aspects^1,2^. As the needs of students evolve, universities have to meet the challenge and introduce changes that respond to these evolving demands. The learning environment encompasses a wide range of domains related to educational, physical, social, and psychological contexts that have a subsequent impact on the professional skills of the students^3–5^. Bonsaken and other authors reported that the perceived quality of the learning environment directly impacted students’ learning and exam results, their satisfaction with the course or study programme, their personal well-being and overall academic achievement^3,6–9^^,^^10,11^.

According to Bloom definition, the educational or learning environment concept can be defined: “the conditions, external stimuli, and forces which may be physical, social, as well as intellectual forces which challenge the individual and influence students’ learning outcomes”^10,10^.

Assessment of the learning environment is important for enabling improvement in the quality of the curriculum. The most often used and most available way of assessing the learning environment assessment is the evaluation of students’ perception of this environment^10^^,^^13^. So far, the Polish learning environment has not been assessed on a large scale and in order to carry out such assessment it is necessary to adapt the appropriate tools. The research will allow these tools to be used later to assess the learning environment of Polish medical schools.

According to the study by Rusticus et al., the learning environment can be divided into four spheres: psychological, social, cultural and physical^14^. Those engaged in the process of education (both the student and the educator) and also the setting have an impact on the whole learning environment^15^. Focusing only on one aspect, such as student’s perspective, may be insufficient for proper/comprehensive assessment of the learning environment^16^. Thus, it is important to conduct studies to determine the mutual influence of different aspects.

Acton and McNeil described the interrelationship between different dimensions of the learning environments such as space, pedagogy and learning^17,18^.

The psychological aspect of the learning environment refers to feelings as well as the preparation for learning and teaching on the part of students, academic staff and other people engaged in education (e.g. administration, technical support, etc.). Meanwhile, from the social perspective, the relationships among these stakeholders and the emotions associated with education and handling matters connected with the education process at university are relevant, as well as the motivation and expectations of both students and educators. Ideally, these expectations should be similar. An extremely important factor is cultural tolerance, especially in the case of learning environments made up of people from different cultural backgrounds. Misunderstandings due to such issues could have a very negative impact on the whole learning environment. Also, one cannot ignore the conditions of the premises where classes are held, which may also have a significant impact on the quality of education, and thus perception of learning environment. As pointed out by McNeil and Borg, it is not only about the halls and rooms where the classes take place, but also about the space available for students to meet, self-study and rest^17^.

Consequently, there is the need to develop a tool for the assessment of the quality of the learning environment within universities. In 1997, Roff et al. developed the Dundee Ready Educational Environment Measure (DREEM) questionnaire^19^, which remained the gold standard for assessment of the quality of the learning environment in medical universities for many years. Over time, more modern questionnaires emerged that raised important issues that do not have their counterparts in DREEM. For this reason, the Johns Hopkins Learning Environment Scale (JHLES) questionnaire has been used increasingly in recent years to assess the learning environments within medical universities worldwide. It is also a newer questionnaire that deals with current issues of safety and inclusion of medical students. In addition, the JHLES questionnaire is based on an earlier in-depth literature review and the criteria used to determine the strength of validity evidence in a systematic review of studies that assessed medical students’ and residents’ perceptions of the learning environment^4,20^

The JHLES is a 28-item assessment tool designed for measuring students’ perceptions of the academic environment. It was designed by Robert B. Shochet, Jorie M. Colbert and Scott M. Wright of the John Hopkins University School of Medicine^11^ The JHLES was developed using a consistent methodology starting in 2012 and a Likert scale is used to gradeits items. Assessing students’ perceptions of the institutional curriculum, environment, and possibilities, as well as their relationships with peers and university staff and their level of involvement in the academic community, is the goal of the JHLES. The JHLES has been modified, translated, and utilized in a few countries so far. In Brazil Damiano et al demonstrated reliability and validity for the JHLES. as feasible option for measuring learning environment in Brazilian medical students^21^. In China the JHLES was utilized to assess medical students’ perceptions of medical school learning environment; the main goal of Zhou et al study was to identify influencing factors for medical students’ perception levels^22^.In Malaysia Tackett et al validated the JHLES and measured the learning environment using two tools: JHLES and DREEM^23,24^. The JHLES has not been translated or validated for Polish conditions until now.

## Materials and methods

The objective here was to translate and adapt the JHLES questionnaire to Polish cultural conditions and validate the Polish version of the tool.

This study was part of a project fully funded by the National Science Centre. The aim of the project was to validate DREEM and JHLES. The validation of the DREEM questionnaire has already been published^25^. The aim of the project was also to validate a more modern questionnaire that is becoming increasingly popular. In addition, there are Items in the JHLES that raise important issues (e.g. 24–26) that have no equivalent in the DREEM, both questionnaires complement each other well. The two validations are not presented in one paper, as most journals have a page or character limit for papers. The project manager decided that this route would be more accessible for future readers. Acquiring adequate research tools is crucial for analyzing the educational environment, our study will enable both methods (the JHLES and DREEM) to be employed to examine the educational environment of Polish medical schools in the future.

Evaluating the learning environment is beneficial because it can reveal how students perceive their surroundings and allow teachers to analyse, plan, and integrate effective teaching strategies to improve it.

To the best of the authors’ knowledge, neither the dental community nor the other medical students involved in this study participated in any other established process for evaluating the learning environment And it was decided that every dental student from the two colleges that were chosen would take part in this investigation.

The objective of the study consisted of the translation, cultural adaptation, and validation of the JHLES questionnaire. As no universal guidelines are available for cultural adaptation of surveys, the authors followed the methodology described in previous studies^26^.

The original JHLES questionnaire is presented in Table 1.

**Table 1:** The original Johns Hopkins Learning Environment Scale (JHLES), with items grouped by subscale.

Scale	Item	Question
1 Community of Peers	1	How connected do you feel to other SOM students?
	2	How supported do you feel in your personal and professional pursuits by other SOM students?
	3	It’s been easy to make friends at the SOM
	4	I feel a sense of community at the SOM
	5	To what extent have you felt a sense of belonging during your time as a student at the SOM?
	6	I’ve encountered an abundance of positive, inspiring role models among fellow students at the SOM
2 Faculty relationships	7	I feel that the SOM faculty I encounter are supportive of my professional goals
	8	I feel that SOM faculty members have taken the time to get to know me
	9	I feel that the SOM faculty I encounter genuinely care about my well-being
	10	I’ve encountered an abundance of positive, inspiring faculty role models at the SOM
	11	There are faculty members that I feel comfortable confiding in when important concerns come up
	12	The faculty advisors in the colleges advisory program are readily accessible and interested in students
3 Academic climate	13	Our medical school’s curriculum allows me to use my preferred learning style
	15	I feel that course exams and assessments test my knowledgeand abilities fairly
	15	I understand the goals and objectives of the SOM curriculum
	16	To what extent do you trust that the institution has fulfilled your needs as a medical student?
	17	The workload during medical school is manageable
4 Meaningful engagement	18	The SOM engages students as meaningful participants
	19	The SOM is flexible and responsive to my needs as a student
	20	I feel that I have a say in decision making about courses and curricular changes
	21	The SOM encourages scholarship and innovation
5 Mentoring	22	I’ve found a mentor in a research field that interests me
	23	I’ve found a mentor in a clinical specialty or discipline that I am passionate about
6 Inclusion and safety	24	I am concerned that students are mistreated at the SOM*
	25	I sense there is discrimination based on gender, race, ethnicity, or sexual identity at the SOM*
	26	I feel concerned at times for my personal safety at the SOM*
7 Physical space	27	The preclinical SOM building has a significant effect on my perception of the learning environment
	28	The work spaces where clinical teaching occurs contributes positively to my sense of the SOM learning environment

*SOM* School of medicine.

*These items were reverse scored.

The JHLES questionnaire was completed by students of all years (first–fifth year) of the faculties of dental medicine at the Medical University of Lublin and the Medical University of Gdansk. The total surveyed population consisted of 650 studentsand their characteristics are given in Table 2. The study was carried out during April–June 2022 and was approved by the Bioethics Committees at the Medical University of Lublin and the Medical University of Gdańsk, as well as by the authorities of both universities. The deans of the various schools of dentistry gave permission for the study to be conducted and the collaborators involved with the schools received written instructions on how to implement the project. One of the authors also carried out the research in both universities.

**Table 2:** Characteristics of the study group after handling missing values n = 597.

Characteristic	N	Distribution^1^
Year of study:	597	
First		114.00 (19.10%)
Second		132.00 (22.11%)
Third		117.00 (19.60%)
Fourth		132.00 (22.11%)
Fifth		102.00 (17.09%)
Sex	597	
Female		466.00 (78.06%)
Male		131.00 (21.94%)
Age [years]	594	22.00 (21.00, 24.00)
University	597	
Gdańsk		272.00 (45.56%)
Lublin		325.00 (54.44%)
Locality	594	
City (> 200 K)		189.00 (31.82%)
City (≤ 200 K)		114.00 (19.19%)
Town (≤ 50 K)		134.00 (22.56%)
Village		157.00 (26.43%)
School endorsement	597	
Exceptional		22.00 (3.69%)
Good		352.00 (58.96%)
Fair		195.00 (32.66%)
Poor		28.00 (4.69%)

^*1*^*n* (%);

^*2*^*Mdn* (*Q1*, *Q3*).

The questionnaire was handed out to the students during their classes. Before starting the survey, each collaborator briefly clarified the aims of the study and the method by which the data will be processed, particularly emphasizing aspects of voluntary participation and anonymity. Sociodemographic data such as age, gender, academic year and origin were also collected^25^.

### Participants and criteria for eligibility

All 418 undergraduate full-time students from the end of the first year through to the fifth year of the Medical University of Lublin and 348 from the Medical University of Gdansk present during the classes when the JHLES and DREEM were administered were invited to participate in this study. Inclusion criteria were to be a dentistry student and give consent for participation in the study. Exclusion criteria were previous participation in a pilot study; lack of consent to participate in the study; and failure to complete the questionnaire twice. Polish medical schools have a 5 years curriculum: the first 2 years are preclinical, followed by 2 years of clinical activity and the last year as mostly hospital activities. Students were informed that 35 days later they would have both the JHLES and DREEM retested. Both questtionnaires were validated simultaneously^25^.

Students who agreed to participate in the survey received no remuneration in any form. The first round of testing lasted approximately 30 min, and the second round about 20 min. To compare both the test and the retest data, students were asked to encode their surveys; surveys were pseudoanonymized, and the students were able to obtain a number from the random number generator. Surveys that had not been encoded or had no matching test/retest surveys were excluded from study^25^.

Sample size selection was based on the generally accepted rule of thumb that there must be at least 5–15 cases per estimated parameter in the confirmatory factor analysis (CFA)^25^.

### Characteristics of the sample

**Figure 1:**
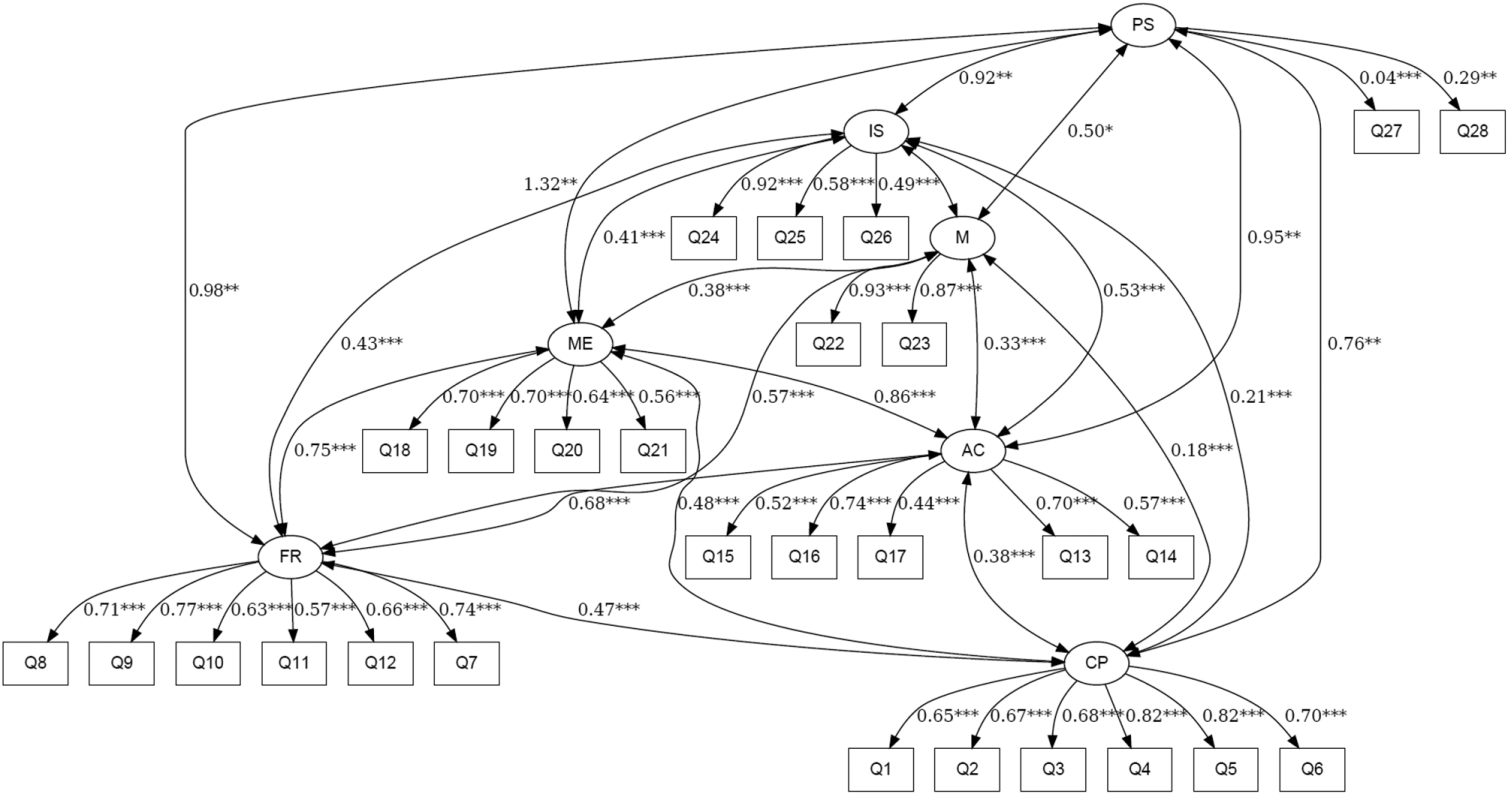
Path diagram of the confirmatory factor analysis model.

The primary sample consisted of 650 undergraduate students (498 females, 152 males) recruited from various departments within the Lublin and Gdańsk universities. Of 650 observations in our sample, the 53 (8.2%) contained 1 to 6 missing values. Within each question, the proportion of missing data did not exceed 1.06% (see Fig. 1 in the Appendices section).

### Translation and transcultural adaptation validity

Firstly, permission to translate and adapt the questionnaires was obtained from the authors. Secondly, the author and two other individuals with a fluent command of English while having Polish as their mother tongue translated the questionnaires into Polish. All three versions were compared and consolidated into a consensus version consistent with the original in terms of terminology, semantics, and concept. A separate consensus was reached for each questionnaire item. Minor changes were introduced to ensure the JHLES was adequate for Polish academic culture. Following the translation of both scales into Polish, they were sent to two native speakers of English who independently translated both questionnaires back into English. In this way, a total of four questionnaires were back-translated, two versions of DREEM and two versions of the JHLES. The two versions of back translations were sent to the original authors so that the final wording of the questionnaires could be determined. The final versions of the questionnaires were translated into Polish by the author and two professional translators and later submitted for consultation and a pilot study with a group of 10–15 members of the Polish Association of Dental Students. Following the consultation and pilot study, final amendments were made to the questionnaires by the Polish authors (Table 3).

**Table 3:** The Polish Johns Hopkins Learning Environment Scale (JHLES)—items grouped by subscale.

Scale	Item	Statement
1 Społeczność rówieśników	1	Czuję się związany(-na) z innymi studentami uczelni medycznej (UM)
	2	Czuję wsparcie od innych studentów UM w realizacji moich zawodowych i osobistych celów
	3	Łatwo jest zawierać przyjaźnie na UM
	4	Czuję się częścią społeczności UM
	5	Jako student(ka) mam poczucie przynależności do UM
	6	Poznałem(-am) wielu pozytywnych i inspirujących studentów, których postrzegam jako wzorce do naśladowania
2 Relacje na Wydziale	7	Czuję, że pracownicy Wydziału, z którymi się zetknąłem/am wspierają moje cele zawodowe
	8	Mam poczucie, że kadra UM poświęciła czas na poznanie mnie
	9	Czuję, że pracownicy Wydziału, z którymi mam kontakt, szczerze troszczą się o moje dobre samopoczucie
	10	Na Wydziale poznałem(-am) wielu pozytywnych i inspirujących pracowników, których postrzegam jako wzorce do naśladowania
	11	Na Wydziale są pracownicy, przy których czuję się komfortowo, zwierzając się w razie pojawiających się trudności
	12	Koordynatorzy/tutorzy są do dyspozycji studentów i są zainteresowani ich sprawami
3 Atmosfera na Uczelni	13	Program nauczania mojej UM pozwala mi na wykorzystanie preferowanego sposobu uczenia się
	14	Mam poczucie, że egzaminy i zaliczenia sprawiedliwie oceniają moją wiedzę i umiejętności
	15	Rozumiem cele i założenia programu studiów UM
	16	Ufam, że UM spełnia moje potrzeby jako studenta medycyny
	17	Obciążenie nauką w trakcie studiów medycznych jest do opanowania
4 Znaczące zaangażowanie	18	UM angażuje studentów jako istotnych/-ne członków/członkinie wspólnoty
	19	UM jest elastyczny i odpowiada na moje potrzeby jako studenta/ki
	20	Mam poczucie, że uczestniczę w podejmowani decyzji dotyczących zmian w przedmiotach i programie nauczania
	21	UM promuje rozwój naukowy i innowacyjność
5 Mentoring	22	Znalazłem(-am) swojego mentora w dziedzinie naukowej, która mnie interesuje
	23	Znalazłem(-am) swojego mentora w specjalizacji klinicznej lub dyscyplinie, która mnie szczególnie pasjonuje
6 Inkluzywność i bezpieczeństwo/Infrastruktura Uczelni	24	Niepokoi mnie, że studenci i studentki są źle traktowani na UM
	25	Mam poczucie, że na UM ma miejsce dyskryminacja ze względu na płeć, rasę, pochodzenie lub orientację seksualną
	26	Czasami czuję się zaniepokojony(-na) własnym bezpieczeństwem na UM
7 Infrastruktura Uczelni	27	Budynki przedkliniczne UM wpływają istotnie na moje postrzeganie środowiska uczenia się
	28	Budynki kliniczne UM przyczyniają się pozytywnie do mojego procesu uczenia się

The cultural adaptation involved adjusting the questionnaire to the Polish academic environment: for example, the term “School of Medicine” was replaced by “Medical University” because in Poland only medical universities exist; “faculty advisors” was replaced by “coordinators/tutors” because they fulfil a similar function in Poland; and due to the dissimilarity of the Polish language, questions were replaced by statements so that the questionnaire could be understandable and grammatically correct. Furthermore, female grammatical forms (Polish: feminatywy), female variants of performers of actions and personal characteristic names were added. Women are named in terms of their titles, fulfilled functions, held positions, practiced professions, nationality, background, faith, convictions, psychological and physical qualities, and performed activities. They represent a class of lexemes with permanent female grammatical gender that consists of syntactically independent nouns. They do not include adjectives or verbs, in the case of which gender is an inflectional category. By adding female grammatical forms, we wanted to address both female and male students^25^.

Questions 1, 2 and 5 were converted into statements in order to standardize the answers on the Likert scale. For each statement, students could answer: strongly agree, agree, disagree, strongly disagree, strongly disagree. If Items 1, 2 and 5 had remained questions, the answers for these three items would have had to be changed to Polish. All changes were approved by the author of the questionnaire and also checked by a professional language corrector.

Items 6 and 10 were also accepted by the author of the original questionnaire and there were no queries during the pilot study or the target study.

### Statistical analysis

Basic descriptive statistics were tabulated with tests for significant differences applied as appropriate. Basic descriptive statistics were used for the study group characteristics and the JHLES results as well as for medical university, gender, and academic year.

Overall JHLES scores were computed by summing across survey items for each scale.

Spearman correlation coefficients were calculated to determine associations between each of the JHLES scales and their respective subcategories or domains.

The reliability of the JHLES questionnaire (both the global scale and the subscales) was evaluated for internal consistency using Cronbach’s α, the value of which is between 0 and 1. This coefficient reflects the homogeneity of the scale, which is the level to which it can be regarded as a measure of a single construct^27^. Following the methodology of Dimoliatis et al., we estimated ‘expected’ α values for the various subscales and compared them with the ‘observed’values^28^.

The significance level of the statistical tests in this analysis was set at α = 0.05 and the normality of the distributions of the variables was analysed using the Shapiro–Wilk test. Numerical variables with distributions that deviated from the normal distribution were reported as the median with quartiles (*Mdn*; *Q1*, *Q3*) and normally distributed variables as the mean and standard deviation (*M*; *SD*).

### Confirmatory factor analysis

The confirmatory factor analysis (CFA) model was specified based on the theoretical framework proposed for measurement to assess students’ perceptions of the medical school learning environment. It was hypothesized that the 28-item JHLES would load onto seven latent factors: community of peers; faculty relationships; academic climate; meaningful engagement; mentoring; inclusion and safety; and physical space. Indicator variables were defined by Likert scale items with values from 1 to 5.

We conducted CFA to test our hypothesized measurement model. A diagonally weighted least squares (DWLS) estimator was performed, which is appropriate for our ordinal data and the optimization method used was NLMINB (non-linear minimization).

The model fit was assessed using several fit indices, including the comparative fit index (CFI), tucker-lewis index (TLI), and root mean square error of approximation (RMSEA), standardized root mean square residual (RSMR).

We also employed Little’s MCAR (missing completely at random) test to evaluate whether the occurrence of missing data in our dataset was completely random^29^.

### Differences between groups

Welch’s t test was used for variables with normal distribution and two groups. The effect size was calculated using Hedges *g*-measure. And the effect obtained was interpreted based on Cohen’s convention^30^.

Welch’s ANOVA test was used for variables with a normal distribution and number of groups above and the effect size was calculated using the omega squared effect size measure. The obtained effect was interpreted based on Field’s convention^31^. Significance between pairs of groups was tested using the Games-Howell test. To account for multiple comparisons between pairs of groups, the significance level was adjusted using the Holm correction.

### Correlation analysis

Spearman’s rank correlation coefficient (rho) was used to measure the strength and direction of association between two numerical variables when at least one variable was not normally distributed; statistical significance was calculated using the algorithm AS 89^32^.

### Statistical environment

Analyses were conducted using the R Statistical language (version 4.1.1; R Core Team, 2021)^33^ on Windows 10 Pro 64 bit (OS build 19045), using the packages *lavaanPlot* (version 0.6.2), *report* (version 0.5.7; Makowski D et al., 2023), *ggstatsplot* (version 0.9.3; Patil I, 2021)^34^, *lavaan* (version 0.6.12)^35^, *gtsummary* (version 1.6.2)^36^, *naniar* (version 1.0.0)^37^, *ggplot2* (version 3.4.0)^38^, *readxl* (version 1.3.1)^39^, *dplyr* (version 1.1.2)^40^, *effectsize* (version 0.8.3)^41^ and *psych* (version 2.1.6)^42^.

### Ethical approval and consent to participate

He study was approved by the Bioethics Committees at the Medical University of Lublin (KE-0254/61/03/2022) and the Medical University of Gdańsk (KE-0254/61/03/2022) as well as by the authorities of both universities. All methods were carried out in accordance with the relevant guidelines and regulations. Informed consent was obtained from all subjects.

## Results

### Handling missing data

The output from Little’s MCAR test was χ^2^ = 1279.222, *p* < 0.001 providing strong evidence against the null hypothesis (data are Missing Completely At Random). This suggested that the occurrence of missing data was systematic and might be related to either observed or unobserved data. The lack of randomness prevented imputation of data, therefore observations with missing values were removed from the dataset. The characteristics of the final sample are presented in Table 2. The overall response rates were 325/418 (78%) and 272/348 (78%) respectively.

### Correlation analysis

As shown by the correlation analysis (Table 4), positive corrected item-subscale and item-total correlations were identified for nearly all item pairs. Correlations between items within each subscale were stronger than those between items from different subscales. Strong positive correlations were identified within the individual subscales, with the exception of Subscale 7, where moderate correlations were identified for Item 27.

**Table 4:** Corrected item-subscale and item-total correlations of Polish Johns Hopkins Learning Environment Scale (JHLES) structure (28 items).

Item	Subscale 1	Subscale 2	Subscale 3	Subscale 4	Subscale 5	Subscale 6	Subscale 7	Global scale
1	0.782**	0.186**	0.190**	0.171**	0.040	0.091*	0.154**	0.420**
2	0.773**	0.253**	0.171**	0.210**	0.083*	0.116**	0.138**	0.453**
3	0.773**	0.255**	0.204**	0.178**	0.069	0.189**	0.140**	0.476**
4	0.774**	0.315**	0.250**	0.343**	0.152**	0.124**	0.206**	0.544**
5	0.727**	0.352**	0.298**	0.385**	0.139**	0.178**	0.219**	0.572**
6	0.718**	0.320**	0.199**	0.274**	0.194**	0.114**	0.127**	0.485**
7	0.377**	0.733**	0.419**	0.455**	0.326**	0.220**	0.166**	0.645**
8	0.263**	0.745**	0.398**	0.485**	0.374**	0.179**	0.183**	0.613**
9	0.273**	0.804**	0.455**	0.503**	0.401**	0.256**	0.213**	0.673**
10	0.281**	0.658**	0.321**	0.332**	0.391**	0.314**	0.155**	0.561**
11	0.245**	0.709**	0.237**	0.284**	0.392**	0.200**	0.109**	0.502**
12	0.237**	0.693**	0.396**	0.449**	0.312**	0.294**	0.150**	0.580**
13	0.246**	0.432**	0.724**	0.535**	0.198**	0.254**	0.136**	0.584**
14	0.170**	0.345**	0.688**	0.359**	0.227**	0.276**	0.196**	0.490**
15	0.165**	0.310**	0.612**	0.348**	0.110**	0.302**	0.111**	0.452**
16	0.249**	0.438**	0.736**	0.523**	0.218**	0.349**	0.157**	0.605**
17	0.198**	0.251**	0.634**	0.311**	0.099*	0.194**	0.003	0.394**
18	0.394 **	0.454**	0.411**	0.712**	0.241**	0.238**	0.269**	0.620**
19	0.200**	0.475**	0.537**	0.812**	0.249**	0.245**	0.160**	0.598**
20	0.246**	0.418**	0.458**	0.757**	0.272**	0.177**	0.100*	0.549**
21	0.247**	0.342**	0.385**	0.646**	0.157**	0.225**	0.309**	0.494**
22	0.153**	0.487**	0.215**	0.315**	0.949**	0.027	0.143**	0.453**
23	0.138**	0.465**	0.236**	0.273**	0.953**	0.014	0.120**	0.435**
24	0.159**	0.335**	0.446**	0.342**	0.061	0.794**	0.177**	0.481**
25	0.110**	0.214**	0.220**	0.152**	0.015	0.824**	0.103*	0.332**
26	0.124**	0.145**	0.184**	0.078	-0.076	0.788**	0.150**	0.268**
27	0.094*	0.047	−0.030	−0.016	0.055	0.024**	0.530**	0.094*
28	0.384**	0.238**	0.216**	0.308**	0.128**	0.226**	0.835**	0.384**

*Correlation significant at the 0.05 level (2-tailed).

**Correlation significant at the 0.01 level (2-tailed).

### Reliability analysis

We estimated the ‘expected’ Cronbach’s α values in the different subscales and compared them with the ‘observed’ values^28^. The overall reliability of the tool was 0,896.

The study was carried out using a test–retest design and therefore intraclass correlation coefficients (ICC) were also determined for the purpose of reliability assessment (Table 5).

**Table 5:** Intraclass correlation coefficients for items in the Johns Hopkins Learning Environment Scale (JHLES) using a two-factor mixed-effects and absolute agreement model.

JHLES items	ICC
1	0.733
2	0.698
3	0.757
4	0.711
5	0.742
6	0.734
7	0.671
8	0.708
9	0.696
10	0.680
11	0.501
12	0.662
13	0.676
14	0.724
15	0.606
16	0.678
17	0.768
18	0.655
19	0.735
20	0.618
21	0.644
22	0.665
23	0.676
24	0.774
25	0.705
26	0.643
27	0.511
28	0.789

### Confirmatory factor analysis

#### Model fit

The model fitted normally after 113 iterations. The number of parameters of final model was 77, with 597 observations.

First, we tested the baseline model, which assumes no relationships among the variables. The chi-square test of the baseline model was statistically significant: χ^2^ (378) = 13,611.73, *p* < 0.001. This result indicated a poor fit of the data to a model assuming no relationships among variables. In other words, it suggested that there were indeed significant relationships among the variables in our dataset, as the likelihood of the observed data giving a model of independence was near zero, *p* < 0.001.

The chi-square test of user model fit was significant: χ^2^ (329) = 526.77, *p* < 0.001. This was because the chi-square test is sensitive to sample size, and for a large sample, even minor discrepancies between the observed and model-implied covariance matrices can result in a significant chi-square value.

Other fit indices also supported the adequacy of the model: the CFI was 0.985, TLI was 0.983, RMSEA was 0.032, (*90%* confidence interval [*90% CI*] = 0.027, 0.037) and SRMR = 0.052. Results are presented in Table 6^43^.

**Table 6:** Fit indices of the confirmatory factor analysis model.

Fit indices	Score
CFI	0.985
TLI	0.983
RMSEA	0.032
SRMR	0.052

*CFI* comparative fit index, *TLI* Tucker-Lewis index, *RMSEA* root mean square error of approximation, *SRMR* standardized root mean square residual.

In summary, our model demonstrated good fit across the different fit indices, suggesting that the proposed factor structure adequately represents the relationships among the observed variables.

#### Parameter estimates

The standardized factor loadings were all significant, demonstrating that each item was significantly related to its respective factor. The loadings and covariance magnitudes, as well as the significance levels, can be found in the path diagram for the fitted CFA model in Fig. 1.

#### JHLES overall score

The distribution of JHLES overall scores for the group of individuals without missing data (*N* = 597) is shown in Fig. 2 (see Fig. 2 in the Appendices section). Visually, the distribution in the form of a histogram and density plot did not deviate from the normal distribution, as evidenced by the result of the Shapiro–Wilk test, *W* = 0.99, *p* = 0.067. The mean overall test score was 89.98 (13.75).

#### JHLES overall score versus year of study

The results of Welch’s ANOVA test showed a significantly higher total JHLES test score for the subjects of the first year of study, *M* = 97.21, *SD* = 13.78, compared to the other years of study, second year, *M* = 90.10, *SD* = 13.33, third year, *M* = 88.91, *SD* = 13.1, fourth year, *M* = 86.30, *SD* = 13.18, andfifth year, *M* = 87.76, *SD* = 12.91. The estimated effect was of moderate magnitude. No significant differences were found between the other groups. For a graphical visualization of the results, see Fig. 3 (see Fig. 3 in the Appendices section).

#### JHLES overall score versus gender

The results of Welch's t-test showed no significant differences between the total score of females, *M* = 89.81, *SD* = 13.67, and males, *M* = 90.61, *SD* = 14.06, *p* = 0.560. The effect obtained was of small size. For a graphical visualization of the results, see Fig. 4 (see Fig. 4 in the Appendices section).

#### JHLES overall score versus university

The results of Welch’s *t*-test showed that the total JHLES core in the Gdańsk University group, *M* = 87.9, *SD* = 13.69 was significantly lower than the total score in the Lublin group, *M* = 91.73, *SD* = 13.56, *p* < 0.001. The effect obtained was of small size (see Fig. 5 in the Appendices section).

#### JHLES overall score versus endorsement

The results of the Welch’s ANOVAtest showed a significantly higher total JHLES test score for the subjects with exceptional endorsement, *M* = 109.14, *SD* = 17.42, compared to the other endorsement levels, of good, *M* = 95.39, *SD* = 10.07, fair, *M* = 81.21, *SD* = 10.22, and poor, *M* = 68.07, *SD* = 11.50.

In addition, the score of the group with good endorsement was significantly higher compared to the groups with fair and poor endorsement. Conversely, the score of the fair group was significantly higher than that of the poor endorsement group. The estimated effect was of large magnitude.

For a graphical visualization of the results, see Fig. 6 (see Fig. 6 in the Appendices section).

#### JHLES overall score versus age

The results of the correlation analysis, *rho* = − 0.21, p = 0.001, showed a significant negative relationship between the JHLES total score and the age of the participants. The total score thus decreased significantly with increasing age.

#### Convergent validity

The convergent validity of the test can be measured by demonstrating a positive correlation between measures of related constructs. In other words, if two scales are related, a subject who scores high on one scale should also score high on the other. Pearson’s *r* (ranging between 1 and −1), is used to estimate correlation by revealing the strength and direction of the relationship between variables. Comparing the total results of JHLES and DREEM surveys, Pearson’s rho amounted to 0.797, (p < 0.001).

## Discussion

The aim of this study was to develop the Polish-adapted version of the JHLES questionnaire. This was the first validation of this tool in Polish conditions as well as the first validation to be performed on such a large group of subjects. Validation was performed using the less common test–retest method and the CFA. Validity analysis also included corrected correlations for the original JHLES structure (item–total and item–subscale), following the recommendations established by Stuive et al^44^. A minimum threshold value of 0.20 was set for the absolute value of the corrected correlations in order to consider it to support construct validity^45^.

The validated questionnaire appears to presentgood internal reliability, as evidenced by its internal consistency and the test–retest consistency indicative of the temporal stability of the results. The overall reliability of the tool was good, near to excellent 0,896 Medium or good consistency (ICC) was demonstrated for individual items, with items 3, 17, 25 and 28 showing good consistency as assessed according to the newest and most restrictive ICC interpretation criteria^46^.

CFA facilitated the development of a model presenting good fitting metrics. Individual items were positively correlated with one another, with the best correlations observed between items within the same subscales. Our study featured the JHLES being used in the largest study group to date (N = 597) and the correlation between the average scores of all items of the JHLES forboth periods was very high (Pearson’s r = 0.790; p < 0.001). Factor analysis is among the most useful methods for studying and validating the internal structure of instruments^47,48^ and CFA specifically addresses the relationships between latent variables or factors and observed measures or indicators (e.g. test items). The chi-square testresults might indicate that the model does. Not fit well, but it is important to remember that this test is highly dependent on sample size. In this instance, the ‘relative chi-square’ (the chi-square statistic divided by its degrees of freedom) was 1.6. Thus, the relative chi-square was less than 5, and the outcome was deemed acceptable.

The fit indices also supported the adequacy of the model: the CFI was 0.985, TLI was 0.983, RMSEA was 0.032, (90%* CI* = 0.027, 0.037) and SRMR = 0.052, The requirements set by Hu and Bentler for an acceptable match (the CFI, TLI values above 0.95, RMSEA values below 0.06 and SRMR below 0.08) are thus met by our fit indices^43^. The results were consistent due to all indices being within acceptable ranges (close to 1 or 0 depending on the situation), which shows that the JHLES model is supported by the data^43^. The correlation coefficients between the JHLES and another tool based on a similar theoretical concept, namely the DREEM, was indicative of the relevance of the newly developed tool. Statistical analysis revealed a significant correlation. Coefficients between the total results of theJHLES and DREEM surveys (p < 0.001). The correlation was positive, i.e. higher total JHLES scores corresponding to higher total DREEM scores. Pearson’s rho amounted to 0.797 also indicating a very strong correlation^25^. This strong correlation coefficients between the DREEM and JHLES total scores suggesting that both questionnaires may measure the same overall concept—the learning environment.

Damiano et al also performed an adaptation of the JHLES and Medical School Learning Environment Scale (MSLES) questionnaires to obtain results with appropriate validity (in terms of content, internal structure, psychometric properties, and relation to other variables). The JHLES instrument has good reliability, with Cronbach α = 0.809. Stability was assessed using test–retest reliability via Pearson’s r and ICCs. The 45-day test–retest comparison resulted in Pearson’s r = 0.757 (p** < **0.001) and a ’single measures ‘ ICC of 0.757 (*p* < 0.001). The MSLES global score showed a significant (*p* < 0.05) and positive correlation with the learning environment, both for school endorsement (*r* = 0.321), and overall learning environment perception (*r* = 0.505). Findings included good test–retest reliability and convergent validity, both of which were significantly correlated with each other, as well as with two questions on overall learning environment perception and school endorsement. The authors had validated the surveys using principal component analysis; the Brazilian version of the questionnaire contained significant differences as compared to the original, with only Factors 3 (Academic Climate), 5 (Physical Space), 6 (Inclusion and Safety), and 7 (Mentoring) maintaining the same structure as the original version. Despite the changes, all subdomains of the Brazilian JHLES and the original versions had similar Cronbach’s α coefficients for comparisons among both the original and the factor revised JHLES and its correlations with each JHLES subdomain^21^.

In a study by Tackett et al, the JHLES showed high internal reliability for the total JHLES score (α = 0.92) and the seven subdomains (α = 0.56–0.85). Each of its seven domains had values of Cronbach’s α within acceptable limits. Similar values were obtained in our study. According to Tackett et al., the corrected item-total correlations for the JHLES showed that all but two items had correlation coefficients above the acceptable level of 0.30^23^. In our study, individual items were positively correlated with one anotheraand the best correlations were observed between items within the same subscales.

Tackett et al. (2015) performed assessment of the DREEM and JHLES in Malaysian medical schools. Their result showed that the DREEM and JHLES scores were highly correlated with one another overall (r = 0.73), with stronger correlations at the Perdana University School of Medicine (r = 0.80) and Cyberjaya University College of Medical Sciences (r = 0.80) compared to Johns Hopkins University School of Medicine (r = 0.64). Our results showed a very strong correlation (r = 0.797). The very strong correlation between the DREEM and JHLES total scores suggests that both questionnaires may measure the same overall construct^24^. In our study, the values of Cronbach’s α for individual scales ranged between 0.554 and 0.901 (Table 7), which translated to good (Scales 1 and 2), acceptable (Scale 3 and 4), and excellent (Scale 5) reliability. The overall reliability of the tool was excellent. ICCs were also determined for reliability assessment. Medium or good consistency was demonstrated for individual items, with Items 17 and 28 showing good consistency as assessed according to the newest and most restrictive ICC interpretation criteria^46^. Our results showed that the correlation was positive, with higher total JHLES scores corresponding to higher total DREEM scores. Pearson’s r was 0.797, indicating a very strong correlation^25^.

**Table 7:** Cronbach’s α coefficients for the different global scales and subscales (‘observed values’ and ‘expected values’) of the Johns Hopkins Learning Environment Scale (JHLES).

JHLES	Items	Cronbach’s α
Global scale	28	0.896 (0.812)
Subscale		
Subscale 1	6	0.870 (0.798)
Subscale 2	6	0.836 (0.797)
Subscale 3	5	0.730 (0.755)
Subscale 4	4	0.743 (0.745)
Subscale 5	2	0.894 (0.894)
Subscale 6	5	0.717 (0.744)
Subscale 7	2	0.023 (0.024)
Global scale—gender		
Women	28	0.898
Men	28	0.892
Year		
1st	28	0.922
2nd	28	0.888
3rd	28	0.887
4th	28	0.880
5th	28	0.874
Global scale—faculty		
Medical University of Lublin	28	0.907
Medical University of Gdańsk	28	0.884

Cronbach’s α ‘expected’ values were calculated using the Spearman–Brown formula. The ‘observed’ values, which were inferior to the ‘expected’ values, appear in bold.

*ITEMS* number of items in the scale or subscale, *n* number of questionnaires (participants), *CASES* number of questionnaires without value lost on which the α coefficients were calculated.

### Limitations

Our study was carried out in two of the ten medical universities in Poland, therefore careful consideration should be paid to the results obtained using the JHLES tool when attempting to generalize these results to other institutions. Furthermore, both the JHLES and DREEM questionnaires were completed on the same day, which might have resulted in the correlation between instruments being stronger than if they were completed at different times. The reason for the lower reliability of Subscale 7 is probably the lack of campus at the Medical University of Gdańsk. Polish students may not consider university buildings as a campus, as well as an important part of the learning environment.

## Conclusions

Our study provided good evidence for the reliability and validity of the Polish version of the JHLES. In conclusion, the Polish-language version of the JHLES questionnaire is a reliable and valid instrument for analysing the learning environment for students, and its factor structure is supported by the data. The validated questionnaire is presented in Table 2. The use of standardized tools for the assessment of learning environments will lead to a better understanding of the local functioning of such environments and facilitate comparison of these environments with those in other countries. Improved functioning of educational environments in Poland is also to be expected.

In future studies, it will be possible to assess the factors causing differences in perception of the learning environment. It will become possible to assess the relationship between the learning environment and the professional development and clinical competencies of students of medical universities in Poland.

## Supplementary Information

Supplementary Figures.

## Data Availability

The datasets generated and/or analysed during the current study are available in Mendeley Data 10.17632/36zpwkbny3.1

## Abbreviations

CFA: Confirmatory factor analysis
CFI: Comparative fit index
DREEM: Dundee ready educational environment measure
GFI: Goodness of fit index
ICC: Intraclass correlation coefficients
JHLES: Johns Hopkins Learning Environment Scale
MIs: Modification indices
PCA: Principal component analysis
RMSEA: Root mean square eror of approximation
SRMR: Standardized root-mean-square residual
*N, n*_*obs*_: Sample size
*n*: Group size
*Mdn*: Median
*Q1*: First quartile
*Q3*: Third quartile
*M*: Mean
*SD*: Standard deviation
*p*: P-value of statistical test
*p*_*Holm-adj*_: P-value with Holm adjustment for multiple comparisons
*90**% CI*: 90% Confidence interval
*F*_*Welch*_: Welch’s ANOVA test statistic
*t*_*Welch*_: Student’s *t*-test statistic
${\widehat{g}}_{hedges}$: Hedges *g* effect size
${\widehat{w}}^{2}$: Omega-square effect size
*W*: Statistic of Shapiro–Wilk test
χ^2^: Chi-square test statistic
*df*: Degrees of freedom
*rho*: The Spearman correlation coefficient
