# Author Correction: Validation of the Polish version of the Johns Hopkins Learning Environment Scale–a confirmatory factor analysis

**DOI:** 10.1038/s41598-024-76289-x

**Published:** 2024-10-22

**Authors:** Dorota Wójcik, Leszek Szalewski, Adam Bęben, Iwona Ordyniec-Kwaśnica, Robert B. Shochet

**Affiliations:** 1 Department of Dental Prosthetics, Medical University of Lublin, Lublin, Poland; 2 Digital Dentistry Lab, Department of Dental and Maxillofacial Radiodiagnostics, Medical University of Lublin, Lublin, Poland; 3 Department of Dental Prosthetics, Medical University of Gdańsk, Gdańsk, Poland; 4 Department of Medicine, Albert Einstein College of Medicine, New York City, NY USA

Correction to: *Scientific Reports *10.1038/s41598-024-61391-x, published online 12 May 2024

The original version of this Article contained errors in Table 3, where items 27 and 28, which constitute Subscale 7—Infrastruktura Uczelni, were incorrectly labelled.

Incorrect:

**Table:** 

Scale	Item	Statement
6 Inkluzywność i bezpieczeństwo/Infrastruktura Uczelni	24	Niepokoi mnie, że studenci i studentki są źle traktowani na UM
	25	Mam poczucie, że na UM ma miejsce dyskryminacja ze względu na płeć, rasę, pochodzenie lub orientację seksualną
	26	Czasami czuję się zaniepokojony(-na) własnym bezpieczeństwem na UM
	27	Budynki przedkliniczne UM wpływają istotnie na moje postrzeganie środowiska uczenia się
	28	Budynki kliniczne UM przyczyniają się pozytywnie do mojego procesu uczenia się

Correct:

**Table:** 

Scale	Item	Statement
6 Inkluzywność i bezpieczeństwo/Infrastruktura Uczelni	24	Niepokoi mnie, że studenci i studentki są źle traktowani na UM
	25	Mam poczucie, że na UM ma miejsce dyskryminacja ze względu na płeć, rasę, pochodzenie lub orientację seksualną
	26	Czasami czuję się zaniepokojony(-na) własnym bezpieczeństwem na UM
7 Infrastruktura Uczelni	27	Budynki przedkliniczne UM wpływają istotnie na moje postrzeganie środowiska uczenia się
	28	Budynki kliniczne UM przyczyniają się pozytywnie do mojego procesu uczenia się

The original Article has been corrected.

