# Supplementary material for: Validation of the Polish version of the Johns Hopkins Learning Environment Scale–a confirmatory factor analysis
Source: Sci Rep. 2024 May 12;14:10843. doi: 10.1038/s41598-024-61391-x (PMC11089035; doi:10.1038/s41598-024-61391-x)

**Figure 1.** Missingness in a data sample (black indicates a missing cell and grey indicates a present cell)
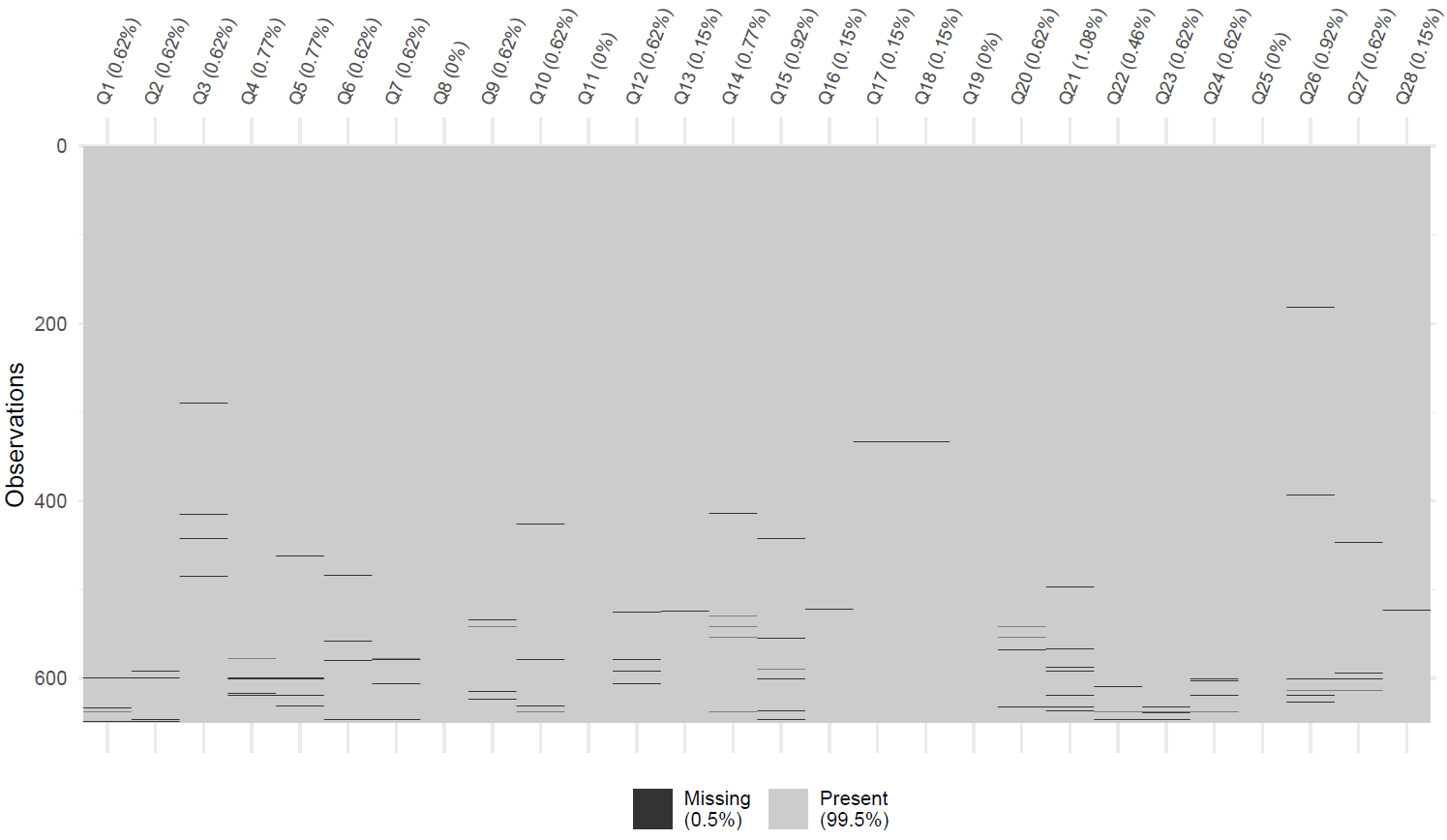


Figure 2. Histogram of JHLES total score with density distribution (the red dashed line indicates the average total score of the sample)


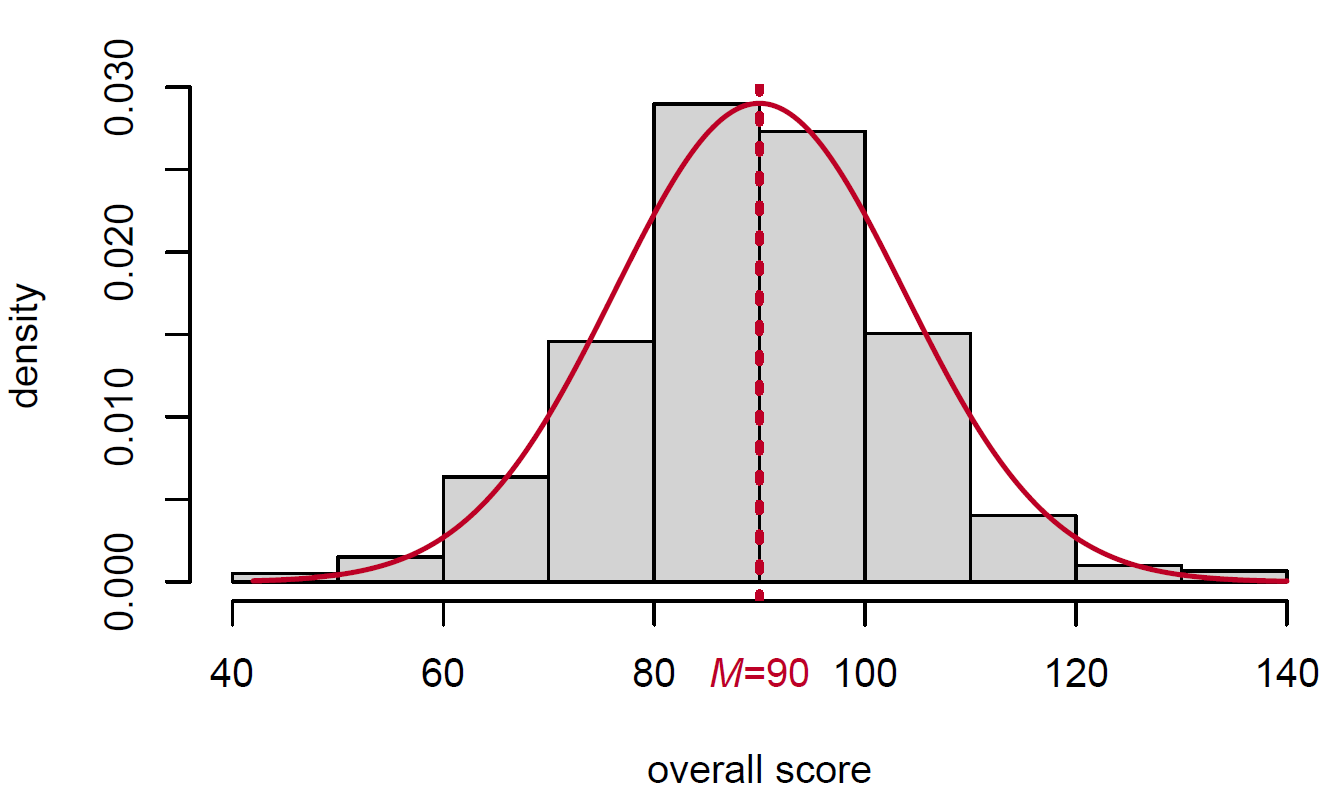


Figure 3. Distribution of JHLES total score by year of study with statistical test evaluation


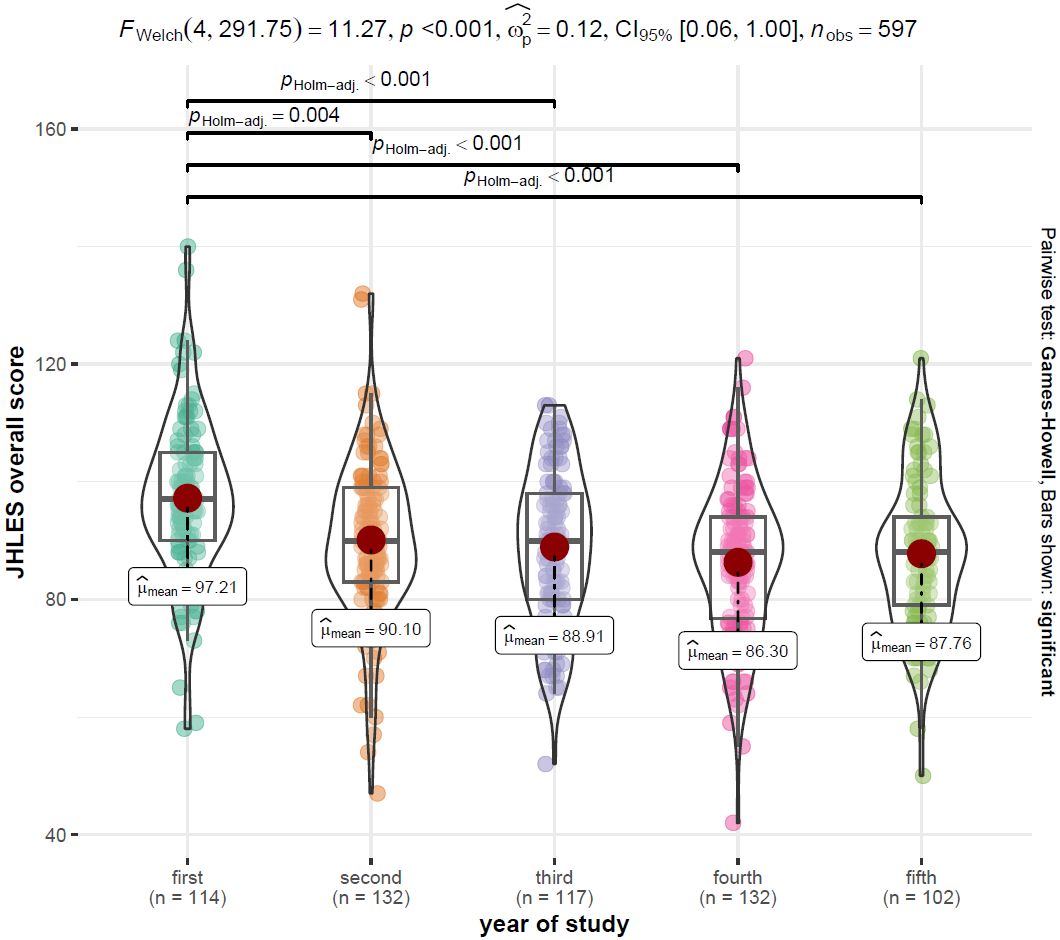


Figure 4. Distribution of JHLES total score by sex with statistical test evaluation


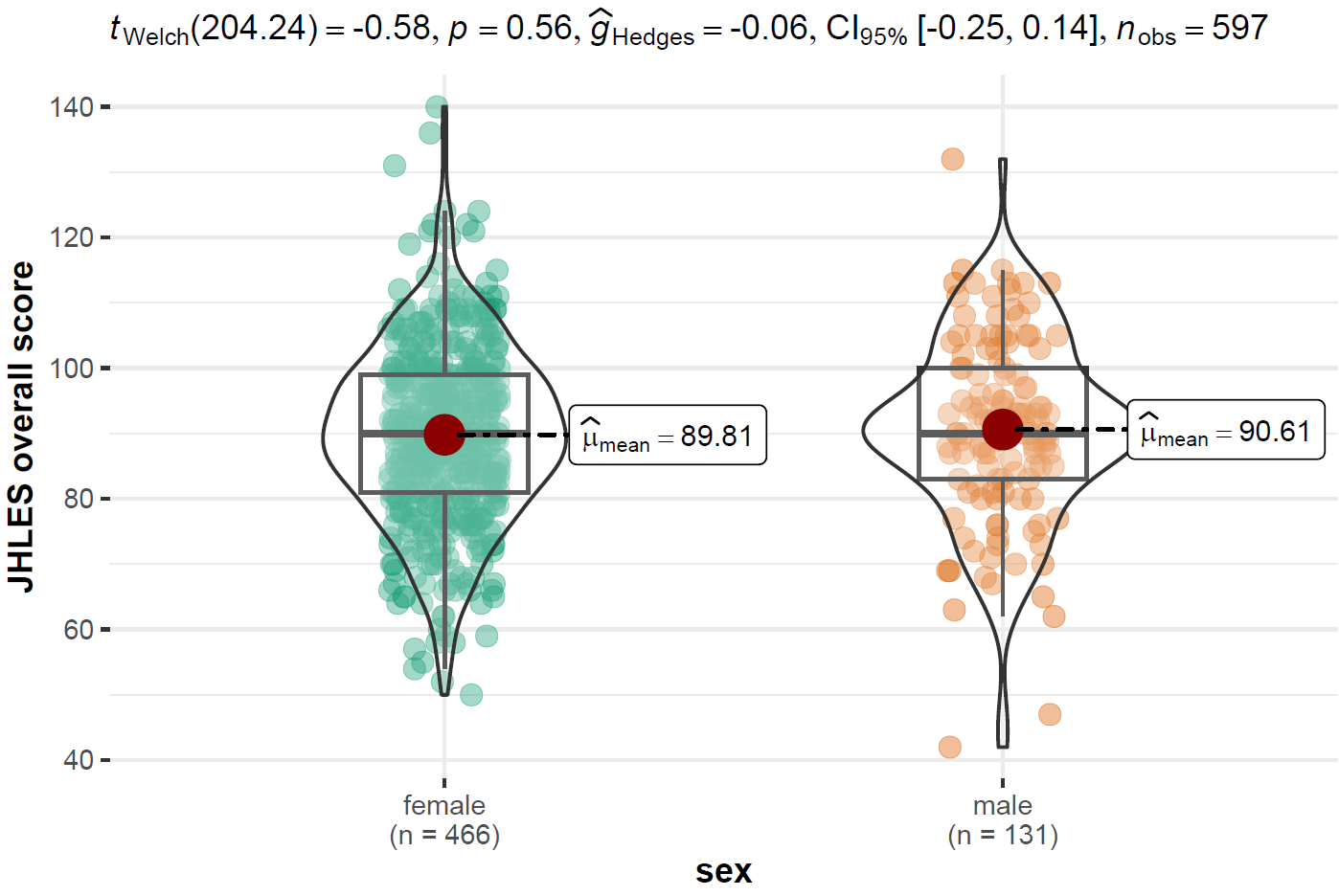


Figure 5. Distribution of JHLES total score by university with statistical test evaluation


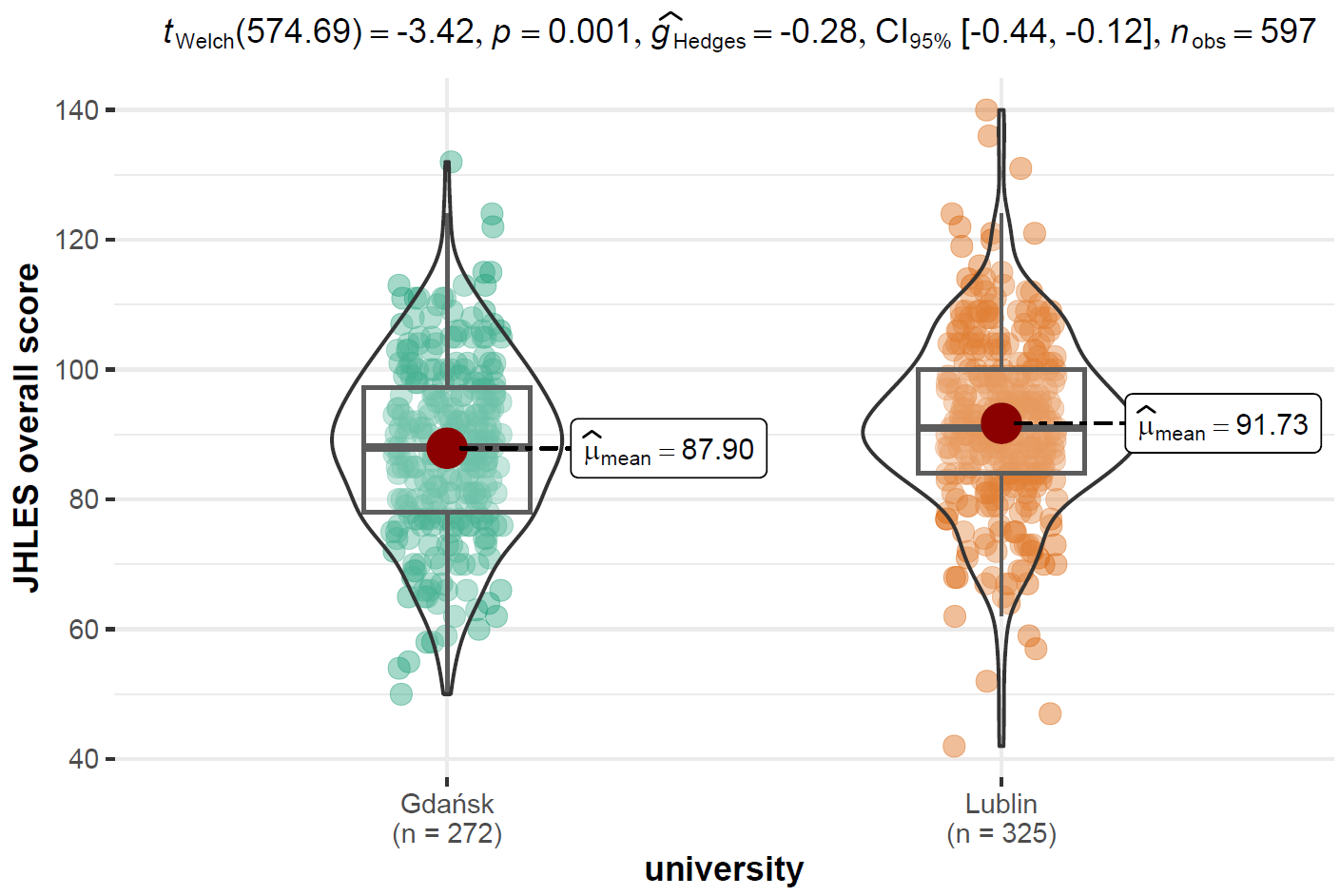


Figure 6. Distribution of JHLES total score by endorsement level with statistical test evaluation


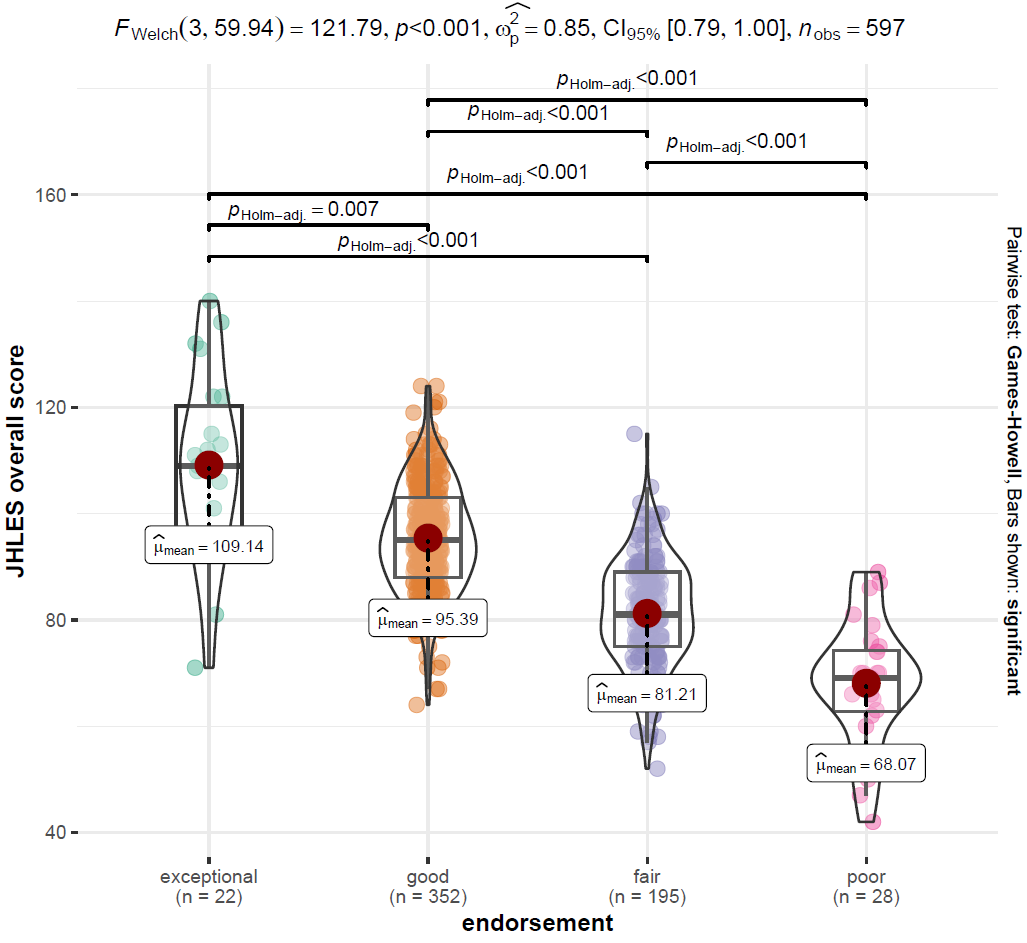

Supplement: Supplementary file 1 — Supplementary Figures. [file 41598_2024_61391_MOESM1_ESM.docx]
